# Integrative analysis of metabolome and transcriptome profiles provides insight into the fruit pericarp pigmentation disorder caused by ‘*Candidatus Liberibacter* asiaticus’ infection

**DOI:** 10.1186/s12870-021-03167-3

**Published:** 2021-08-25

**Authors:** Feiyan Wang, Yunli Wu, Wen Wu, Yongjing Huang, Congyi Zhu, Ruimin Zhang, Jiezhong Chen, Jiwu Zeng

**Affiliations:** 1 Key Laboratory of South Subtropical Fruit Biology and Genetic Resource Utilization & Guangdong Province Key Laboratory of Tropical and Subtropical Fruit Tree Research, Institute of Fruit Tree Research, Guangdong Academy of Agricultural Sciences, 510640 Guangzhou, China; 2 College of Horticulture, South China Agricultural University, 510642 Guangzhou, China

**Keywords:** ‘Shatangju’, Pigment, Carotenoid, Flavonoid, Metabolome, Transcriptome, *Candidatus Liberibacter* asiaticus

## Abstract

**Background:**

Mandarin ‘Shatangju’ is susceptible to Huanglongbing (HLB) and the HLB-infected fruits are small, off-flavor, and stay-green at the maturity period. To understand the relationship between pericarp color and HLB pathogen and the effect mechanism of HLB on fruit pericarp coloration, quantitative analyses of HLB bacterial pathogens and carotenoids and also the integrative analysis of metabolome and transcriptome profiles were performed in the mandarin ‘Shatangju’ variety with four different color fruits, whole green fruits (WGF), top-yellow and base-green fruits (TYBGF), whole light-yellow fruits (WLYF), and whole dark-yellow fruits (WDYF) that were infected with HLB.

**Results:**

the HLB bacterial population followed the order WGF > TYBGF > WLYF > WDYF. And there were significant differences between each group of samples. Regarding the accumulation of chlorophyll and carotenoid, the chlorophyll-a content in WGF was the highest and in WDYF was the lowest. The content of chlorophyll-b in WGF was significantly higher than that in other three pericarps. There were significant differences in the total content of carotenoid between each group. WGF and TYBGF pericarps were low in phytoene, γ-carotene, β-cryptoxanthin and apocarotenal, while other kinds of carotenoids were significantly higher than those in WDYF. And WLYF was only short of apocarotenal. We comprehensively compared the transcriptome and metabolite profiles of abnormal (WGF, TYBGF and WLYF) and normal (WDYF, control) pericarps. In total, 2,880, 2,782 and 1,053 differentially expressed genes (DEGs), including 121, 117 and 43 transcription factors were identified in the three comparisons, respectively. The qRT-PCR confirmed the expression levels of genes selected from transcriptome. Additionally, a total of 77 flavonoids and other phenylpropanoid-derived metabolites were identified in the three comparisons. Most (76.65 %) showed markedly lower abundances in the three comparisons. The phenylpropanoid biosynthesis pathway was the major enrichment pathway in the integrative analysis of metabolome and transcriptome profiles.

**Conclusions:**

Synthesizing the above analytical results, this study indicated that different color pericarps were associated with the reduced levels of some carotenoids and phenylpropanoids derivatives products and the down-regulation of proteins in flavonoids, phenylpropanoids derivatives biosynthesis pathway and the photosynthesis-antenna proteins.

**Supplementary Information:**

The online version contains supplementary material available at 10.1186/s12870-021-03167-3.

## Background

Mandarin ‘Shatangju’ (*Citrus reticulata* cv. ‘Shatangju’) is one of the superior native citrus varieties in Guangdong Province, China. Its fruits are very popular because of its soft and delicate taste, smell, thin pericarp and some other advantageous fruit characteristics [1–3]. However, mandarin ‘Shatangju’ (*Citrus reticulata* cv. ‘Shatangju’) is susceptible to Huanglongbing (HLB), caused by *Candidatus Liberibacter* spp., which has greatly affected ‘Shatangju’ plantations. The typical symptoms of HLB-infected fruits are that the fruits are small, lopsided, and off-flavor, and their stylar end remains green at the maturity period, such as in sweet orange and mandarin ‘Shatangju’ [4, 5]. However, some fruits of one HLB-infected ‘Shatangju’ tree are similar to the fruits of HLB-uninfected tree in terms of quality and appearance [6]. In previous research reports, the influence of HLB infection on citrus fruit yield and quality including internal and external quality have been reported [7]. But the influencing mechanism of HLB infection was not well clarified.

Carotenoids and flavonoids are the main metabolites related to the coloration of citrus fruit. Citrus flavonoids encompass several subgroups of flavonoids, including flavanones (naringin and hesperidin) and O-polymethoxylated flavones (PMFs, nobiletin and tangeretin), especially in sweet orange (*Citrus sinensis* L. Osbeck) and mandarin (*Citrus reticulata* Blanco) [8]. Citrus employs multigene families to control each step of the flavonoid biosynthesis pathway, resulting in a highly complex network. The phenylpropanoid pathway is the upstream stage of the whole process of citrus-derived flavonoid synthesis. P-coumaroyl-CoA, as the precursor, is synthesized in three steps by passing through phenylalanine ammonia-lyase (PAL), cinnamate 4-hydroxylase (C4H), and 4-coumarate: coenzyme A ligase (4CL) in turn. All intermediates and enzymes play crucial roles in the regulation of subsequent reactions in flavonoid biosynthesis [9, 10]. The genes promote the production of key flavonoid metabolites, such as naringenin, hesperetin, eriodictyol and sakuranetin. The constituent enzymes mainly include chalcone synthase (CHS), chalcone isomerase (CHI), flavanone 3-hydroxylase (F3H), flavonoid 3’-hydroxylase (F3’H), isoflavone synthase (IFS), flavonol synthase (FLS), dihydroflavonol-4-reductase (DFR), leucoanthocyanidin reductase (LAR), anthocyanidin reductase (ANR), anthocyanidin synthase (ANS) and UDP- glycosyltransferases (UGT) [11–13]. Although the citrus homologs (*CHS*, *CHI, F3H*, *FLS* and *UGT*) are cloned and characterized, it is still unclear the effect of HLB on these genes in citrus fruit pericarps.

The carotenoid biosynthetic pathway is one of the important metabolic pathways in citrus. Its composition and content of carotenoid not only directly determine the color appearance of fruits, but also affect the nutrition. The carotenoids in citrus mainly include α-carotene, β-carotene, violaxanthin, antheraxanthin, lutein, zeaxanthin, α-cryptoxanthin, β-cryptoxanthin, phytoene, lycopene and so on [14]. Depending on the cumulative difference of carotenoid, citrus varieties can be broadly divided into four categories: categories I that are rich in β-cryptoxanthin (such as *Citrus reticulata* Blanco), categories II that are rich in violaxanthin (such as *Citrus sinensis* Osbeck), categories III that are rich in phytoene (*Citrus paradisi* Macf) and categories IV that are low in carotenoids (*Citrus limon* L. Burm) [15, 16]. The carotenoids not only are influenced by genetic factors, but also are affected by a variety of environmental factors, such as temperature, light and disease. The accumulation of β-cryptoxanthin in mandarin fruits was due to the high expression of upstream genes and the low expression of downstream hydroxylase genes [17]. The expression of carotenoid cleavage oxygenase (CCD) gene was significantly correlated with the accumulation of violaxanthin [18]. When the expression level of (E/Z)-phytoene, phytofluene and lycopene related synthase gene were high and the lycopene cyclase gene were low, the accumulation of lycopene and its precursors would increase substantially [19]. Although the related genes of carotenoid have been basically perfected, the effect mechanism of Huanglongbing on carotenoid still needs to be further analyzed.

Transcriptome analysis of citrus has successfully been used for identifying genes involved in the synthesis and signal transduction of photosynthesis, carbohydrate metabolism [20], jasmonic acid biosynthesis [21] and flavonoid biosynthesis [22]. Transcriptome profiling was applied to analyze the gene expression of the leaf [22], root [23] and pulp samples [24] that were related to HLB. However, transcriptome analysis of different colored pericarps of mandarin ‘Shatangju’ fruits associated with HLB was lacking. The application of metabolomics is widely used to investigate various fruits or vegetables and has significantly facilitated the identification of active metabolites and correlated metabolic pathways in various plants [25, 26]. The combination of metabolomics and transcriptomics would help to reveal the biosynthetic mechanisms of key metabolic pathways. Therefore, it is feasible to analyze the carotenoid and flavonoid biosynthesis pathways in citrus by applying these two omics technologies.

The fruits color, as an important appearance and quality character of citrus fruits, has been widely studied [12, 27]. In this study, the affected mechanism of fruit color by HLB was further clarified in WGF, TYBGF and WLYF fruits by combination of metabolomics and transcriptomics data. According to the metabolome results, the levels of some flavonoid compounds were drastically decreased and even could not be detected, whereas some flavonoids showed high accumulation in WGF, TYBGF and WLYF fruits compared with that in WDYF fruits. Fourteen kinds of carotenoids were identified and quantified in the four kinds of mandarin ‘Shatangju’ pericarps. Correlation network were mapped to highlight the regulatory genes associated with flavonoid and carotenoid metabolites by combination of genes and metabolites. These findings provide new insight into the molecular mechanisms associated with the biosynthesis and regulation of metabolites under the influence of HLB in mandarin ‘Shatangju’ pericarps and emphasize the usefulness of the integration method for understanding this process.

## Methods

### Plant materials

*Citrus reticulata* cv. ‘Shatangju’ for this study were grown at the citrus research orchard at Institute of Fruit Tree Research, Guangdong Academy of Agricultural Sciences, Guangzhou, Guangdong Province, China (E 113°21’59”, N 23°9’15”). We chose ten HLB-infected mandarin ‘Shatangju’ trees that were selected from twenty-two trees by PCR. All trees were infected by *Candidatus Liberibacter* asiaticus and fresh fruits were collected from ten HLB-infected trees and mixed together in January 2019. all fruits were classified into four types according to the coloration: whole green fruits (WGF), top-yellow and base-green fruits (TYBGF), whole light-yellow fruits (WLYF), and whole dark yellow fruits (WDYF). The color was detected by a colorimeter (Minolta CR-300, Konica Minolta Investment Ltd, Shanghai, China). Three biological replicates were collected per sample, each with 60 fruits randomly collected from 10 HLB-infected mandarin ‘Shatangju’ trees. The fruits were transported back to the laboratory, and the pericarps were carefully excised with scalpels, collected, frozen in liquid nitrogen, roughly ground, and kept at -80 °C for further research.

### Quantification of *Candidatus liberibacter* asiaticus by qRT-PCR

Total DNA of 100 mg pericarps for each sample was extracted by the CTAB method [28]. Specific primers for 16S rDNA, HLBasf (5’-TCGAGCGCGTATGCAATACG-3’)/HLBasr (5’-GCGTTATC CCGTAGAAAAAGGTAG-3’), and a probe, HLBp (5’(FAM)-AGACGGGTGAGTA ACGCG-(BHQ-1)-3’), were used for the qRT-PCR [29]. The vector pMD-18T was constructed by adding the 16 S rDNA of *Candidatus Liberibacter* asiaticus, about 1200 bp. The primers of 16 S rDNA were P1(5-’GCGCGTATGCAATACGAGCGGC-3’)/P2(5’-GCCTCGCGACTTCGCAACCCAT-3’). The recombinant plasmid pMD-18T-16 S rDNA was used for constructing a standard curve and equation relating the plasmid copy number and CT value [30]. According to the equation, the HLB bacterial population of *Candidatus Liberibacter* asiaticus in pericarps was calculated. The plasmid copy number was calculated based on the equation ‘(amount in ng× 6.022 × 10^23^)/(length in bp×1 × 10^9^ ng/g ×650 g/mole of bp) [30], where length = 3829 bp’. The amplification system consisted of 11.2 µL of probe qRT-PCR premix system (Takara, Ishiyama, Japan), 0.4 µL each of 10 µmol/L forward and reverse primer, 2 µL of cDNA template, and sterile distilled water to a total volume of 20 µL, and the protocol was 95 ℃ for 20 s followed by 40 cycles at 95 ℃ for 1 s and 60 ℃ for 20 s in a StepOnePlus Real-Time PCR System (Applied Biosystems, USA). All reactions were performed in triplicate, and each run contained one negative and one positive control.

### Exaction and detection of chlorophyll and carotenoid

The exaction and detection method of chlorophyll was acetone method by spectrophotometer. The exaction protocol of carotenoids was described as follows: plant materials (100 mg fresh weight) were frozen in liquid nitrogen, ground into powder, and extracted with n-hexane: acetone: ethanol (2:1:1, V/V/V, Merck, Darmstadt, Germany). The extract was vortexed (30 s), ultrasound-assisted extraction was carried out for 20 min at room temperature, and centrifugation was performed (12,000 rpm for 5 min). The supernatants were collected, and the steps above were repeated. The supernatant was collected twice, then evaporated to dryness under a nitrogen gas stream, and reconstituted in 75 % methanol (V/V, Merck, Darmstadt, Germany). The solution was centrifuged, and the supernatant was collected for LC-MS analysis. Eighteen kinds of carotenoids were used for quantitative analysis. All of the carotenoid standards were purchased from Olchemim Ltd. (Olomouc, Czech Republic) and Sigma (St. Louis, MO, USA). Acetic acid was obtained from Sinopharm Chemical Reagent Co., Ltd. (Shanghai, China). The stock solutions of standards were prepared at a concentration of 10 mg/mL in ACN. All stock solutions were stored at -20 °C. The stock solutions were diluted with ACN to working solutions before analysis.

### UHPLC conditions and APCI-Q TRAP-MS/MS for carotenoids

The detection protocol of carotenoids by UHPLC was described as follows: the carotenoid extracts were analyzed using an LC-ESI-MS/MS system (UHPLC, Exion LC™ AD; MS, Applied Biosystems 6500 Triple Quadrupole). The analytical conditions were as follows, HPLC: column, YMC C30 (3 μm, 2 mm×100 mm); solvent system, acetonitrile(ACN): methanol (3:1, V/V) (containing 0.01 % 2;6-Di-Tert-Butyl-4-Methylphenol, BHT): methyl tert-butyl ether (containing 0.01 % BHT); gradient program, 85:5 (V/V) at 0 min, 75:25 (V/V) at 2 min, 40:60 (V/V) at 2.5 min, 5:95 (V/V) at 3 min, 5:95 (V/V) at 4 min, 85:15 (V/V) at 4.1 min, and 85:15 (V/V) at 6 min; flow rate, 0.8 mL/min; temperature, 28 °C; and injection volume: 5 µL. The effluent was alternatively connected to an ESI-triple quadrupole-linear ion trap (Q TRAP)-MS.

An API 6500 Q TRAP LC/MS/MS System was equipped with an APCI Turbo Ion-Spray interface, operated in positive ion mode and controlled by Analyst 1.6.3 software (AB Sciex, USA). The APCI source operation parameters were as follows: ion source, turbo spray; source temperature, 350 °C; curtain gas (CUR), 25.0 psi; and collision gas (CAD), medium. DP (declustering potential) and CE (collision energy) for individual MRM transitions was performed with further DP and CE optimization. A specific set of MRM transitions were monitored for each period according to the carotenoids eluted within this period.

### Extraction and qualitative and quantitative analysis of metabolites

The extraction and separation of metabolites of mandarin ‘Shatangju’ fruit pericarps were reported previously [27]. In brief, ca. 100 mg fine powder of per sample was extracted with 70 % methanol overnight at 4 ℃. Then, the sample was centrifuged and filtered to obtain the extract of pericarps before LC-MS analysis. The qualitative and quantitative analyses of metabolites were performed by ESI-Q TRAP-MS/MS by a professional metabolomics company (Metware Biotechnology Co., Ltd., Wuhan, China). The significantly changed metabolites (SCMs) were filtered according to | Log_2_ (Fold Change) | ≥1 and *p*-value < 0.05. The specific procedures refer to Wang et al [27].

### RNA extraction and illumina sequencing

The isolation and purification of RNA, construction of cDNA libraries, and sequencing were completed by a professional transcriptome sequencing company (Novogene Bioinformation Technology Co., Ltd., Beijing, China). The filtered reads were mapped to the *Citrus sinensis* genome(https://www.citrusgenomedb.org/organism/Citrus/sinensis) using HISAT2 software [31]. Reads Per Kilo Base per Million mapped reads (FPKM) was used as an indicator for gene and transcript level quantification [32]. The differentially expressed genes (DEGs) were determined by the absolute value of log_2_FoldChange (log_2_FC) greater than 1 and false discovery rate (FDR) ≤ 0.05. All DEGs were analyzed by gene ontology (GO) [33] and Kyoto Encyclopedia of Genes and Genomes (KEGG) enrichment using KOBAS software [34]. The transcription factors were predicted by ITAK software [35].

### Quantitative real-time PCR (qRT-PCR) validation

Total RNA extraction was carried out by the polysaccharide polyphenol plant Total RNA Fast Extraction Kit (BioTeke, Beijing, China). Reverse transcription was performed using a PrimeScript II First Strand cDNA Synthesis Kit (Takara, Ishiyama, Japan). Twelve genes were selected for qRT-PCR with specific primers (Supplemental Table S1). The qRT-PCR was performed with a StepOnePlus Real-Time PCR System (Applied Biosystems, USA) using a TB Green SYBR Kit (Takara, Ishiyama, Japan). The amplification system consisted of 10.4 µL of TB Green SYBR Premix System II, 0.4 µL each of 10 µmol/L forward and reverse primer, 2 µL of cDNA template, and sterile distilled water to a total volume of 20 µL. The amplification program was 95 ℃ for 30 s, followed by 40 cycles of 95 ℃ for 5 s and 60 ℃ for 30 s. The 2^−∆∆ct^ method was performed to analyze relative quantitative data with a reference gene, *β-actin*. Three technical replicates were carried out for each sample to ensure reproducibility and reliability.

### Integrative analysis of metabolome and transcriptome data

Carotenoids and the SCMs in the metabolome and DEGs in the transcriptome were chosen for integrative analysis. SCMs were filtered on the basis of variable importance in the projection (VIP) greater than 1, *p*-value less than 0.05, and absolute values of Log_2_FC greater than 1 for correlation analysis. Pearson correlation coefficients and *p*-values were calculated for carotenoids and SCMs and DEGs data integration using the Spearman method.

### Statistical analysis

SPSS 22.0 was performed for statistical analysis. Data are presented as means ± standard deviations (SD). The levels of statistical significance were analyzed by the least significant difference (*p*-value < 0.05).

## Results

### Phenotypic analysis of fruit appearance

**Fig. 1:**
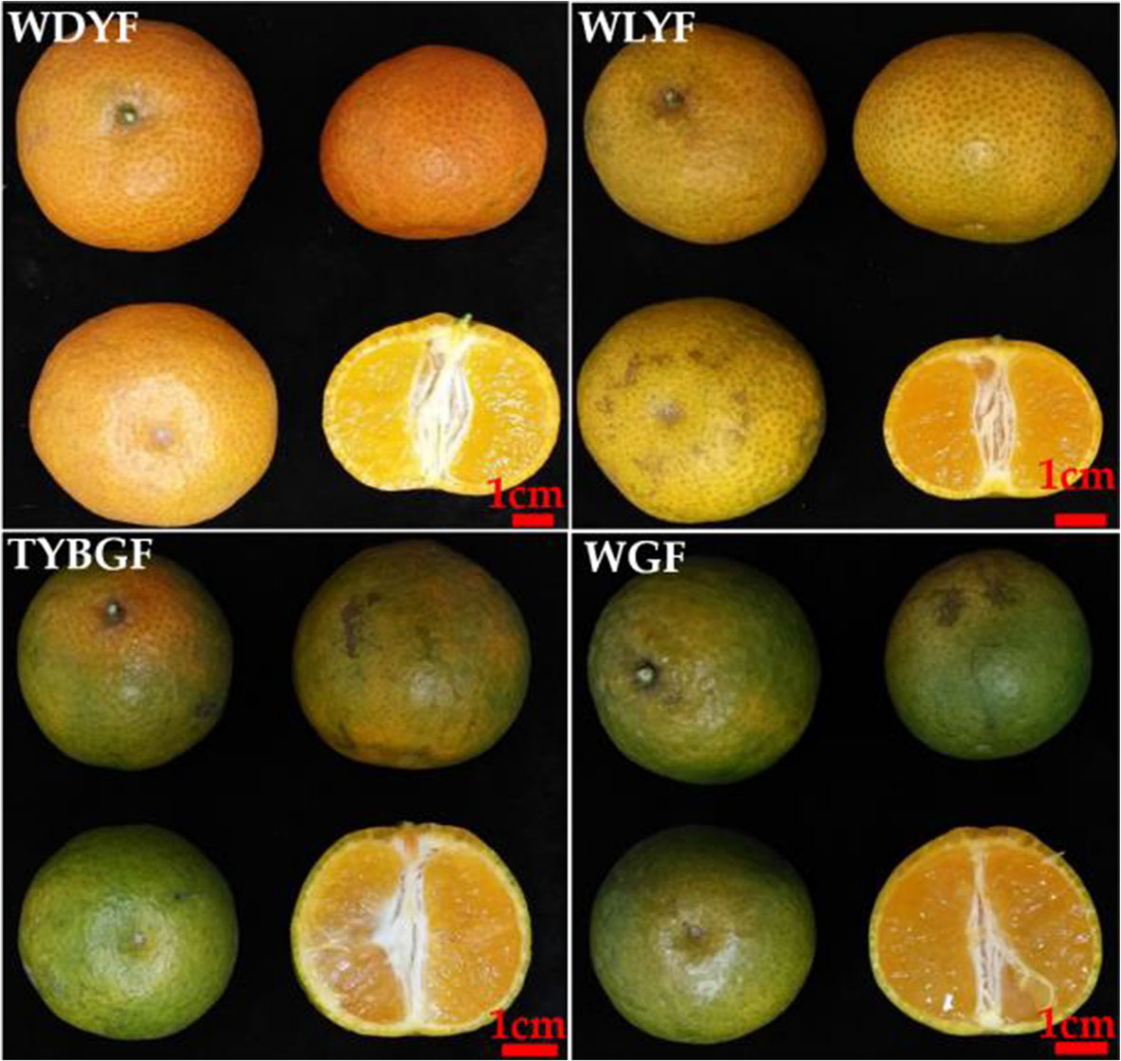
Phenotypes of the four-color types of fruits from HLB-infected mandarin ‘Shatangju’ trees. WDYF (CK): whole dark yellow fruits; WLYF: whole light-yellow fruits; TYBGF: top-yellow and base-green fruits; WGF: whole green fruits

The fruits used in the present study all were harvested at the fruit maturity. The development of mandarin ‘Shatangju’ fruits is very rapid at the last maturity period. The fruits quickly enlarge and color so that they reach their final coloration and size in two months. However, if the trees are infected by the HLB-associated pathogen, *Candidatus Liberibacter* asiaticus, the fruit development will be affected. Herein, we grouped the fruits collected from HLB-infected trees at the mature stage into four types, namely, whole green fruits (WGF), top-yellow and base-green fruits (TYBGF), whole light-yellow fruits (WLYF), and whole dark-yellow fruits (WDYF, CK), based on their coloration and size (Fig. 1, Supplemental Table S2a). Both WGF and TYBGF fruits can’t entirely turn dark-yellow in appearance. The WGF fruits were whole green (H_0_ = 104.49, CI (color index, a/b ratio) = -0.29) and the smallest (32.68 × 30.71 mm). TYBGF fruits were bigger (35.62 × 33.77 mm), and the color of the pedicel parts was lighter-yellow (H_0_ = 100.3, CI=-0.18) than that in WGF fruits. The vertical and horizontal diameters of WGF and TYBGF fruits both were significantly lower than that of WDYF and the WGF were lowest (Supplemental Table S2a). The WLYF fruits could not completely develop dark-yellow pericarp so that they all were light-yellow (H_0_ = 72.72, CI = 0.31), and the WDYF fruits were the darkest yellow fruits (H_0_ = 55.12, CI = 0.70) in all samples, but there was no significant difference in the vertical and horizontal diameters between the WLYF and WDYF fruits (Supplemental Table S2a).

### Quantitation of the HLB bacterial population in pericarps

**Fig. 2:**
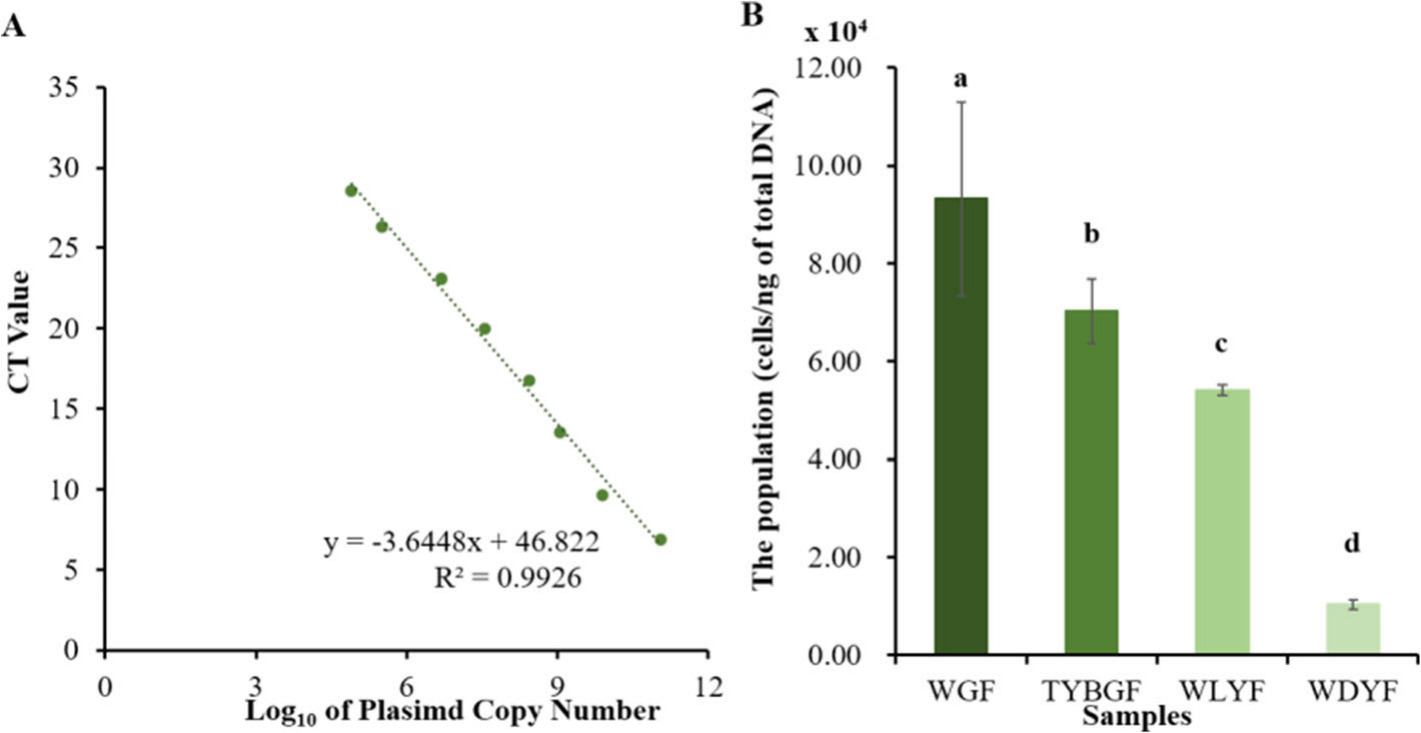
Quantitation of *Candidatus Liberibacter* asiaticus in all samples. **A**: the standard curve between plasmid copy number and CT value of qRT-PCR of the recombinant plasmid; **B**: the HLB bacterial population in the four types of fruit pericarps

By constructing the recombinant plasmids of 16 S rDNA and establishing a standard curve between plasmid copy number and CT value of qRT-PCR, the equation, y=-3.6448x + 46.822 (*R*²=0.9926) was established (Fig. 2 A). According to the equation and mean Ct values of qRT-PCR of HLB bacterial pathogen in the four types of pericarps, the population of HLB bacterial pathogens in per nanogram of total DNA were calculated. The results show that the population of *Candidatus Liberibacter* asiaticus in WGF, TYBGF, WLYF and WDYF fruit pericarps were 9.33 × 10^4^, 7.03 × 10^4^, 5.41 × 10^4^, and 1.03 × 10^4^ cells/ng of total DNA, respectively (Fig. 2B). According to significant difference analysis, WGF, TYBGF and WLYF fruit pericarps were all significantly higher than WDYF fruit pericarps in the population of *Candidatus Liberibacter* asiaticus. The WGF had the highest population of HLB bacterial pathogens and also was most seriously affected in phenotype.

### Quantitative analysis of carotenoid in all samples

**Fig. 3:**
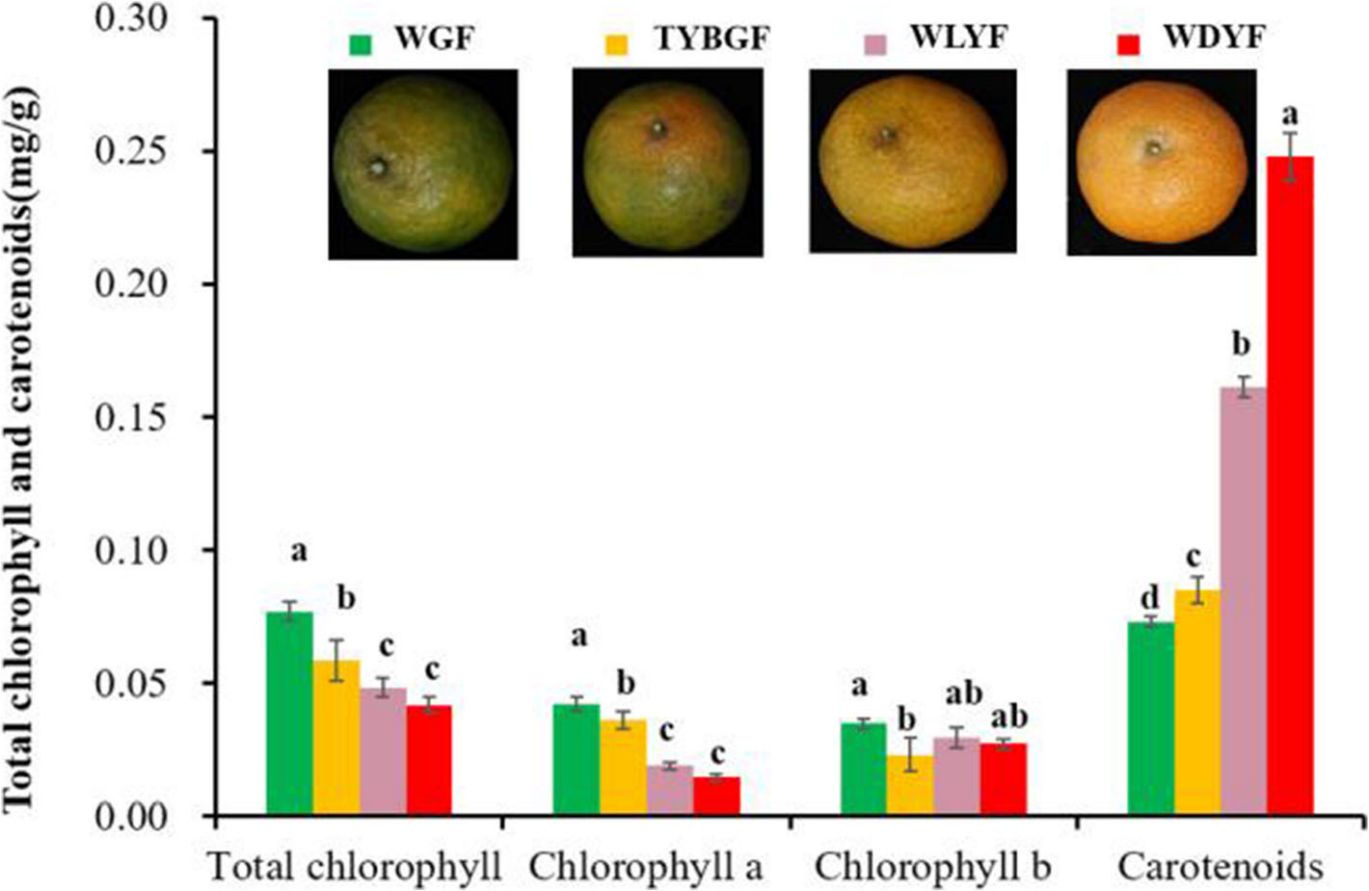
Total content of chlorophyll and carotenoid in all pericarps

The total contents of chlorophyll in WGF, TYBGF, WLYF and WDYF showed a gradually decreasing trend, and was 7.69 × 10^− 2^, 5.85 × 10^− 2^, 4.83 × 10^− 2^ and 4.17 × 10^− 2^ mg/g, respectively (Fig. 3). The content of chlorophyll a in WGF was the highest, followed by TYBGF, with no significant difference between them, and the content of chlorophyll a in WDYF was the lowest. The content of chlorophyll b in WGF was the highest, 3.48 × 10^− 2^ mg/g, and showed significant difference with other three types of fruit pericarps. There was no significant difference among other three types of fruit pericarps. Among the four types of fruit pericarps, there were significant differences in the total content of carotenoid. WGF, TYBGF, WLYF and WDYF were 7.31 × 10^− 2^, 8.47 × 10^− 2^, 1.61 × 10^− 1^ and 2.48 × 10^− 1^ mg/g (Fig. 3).

**Fig. 4:**
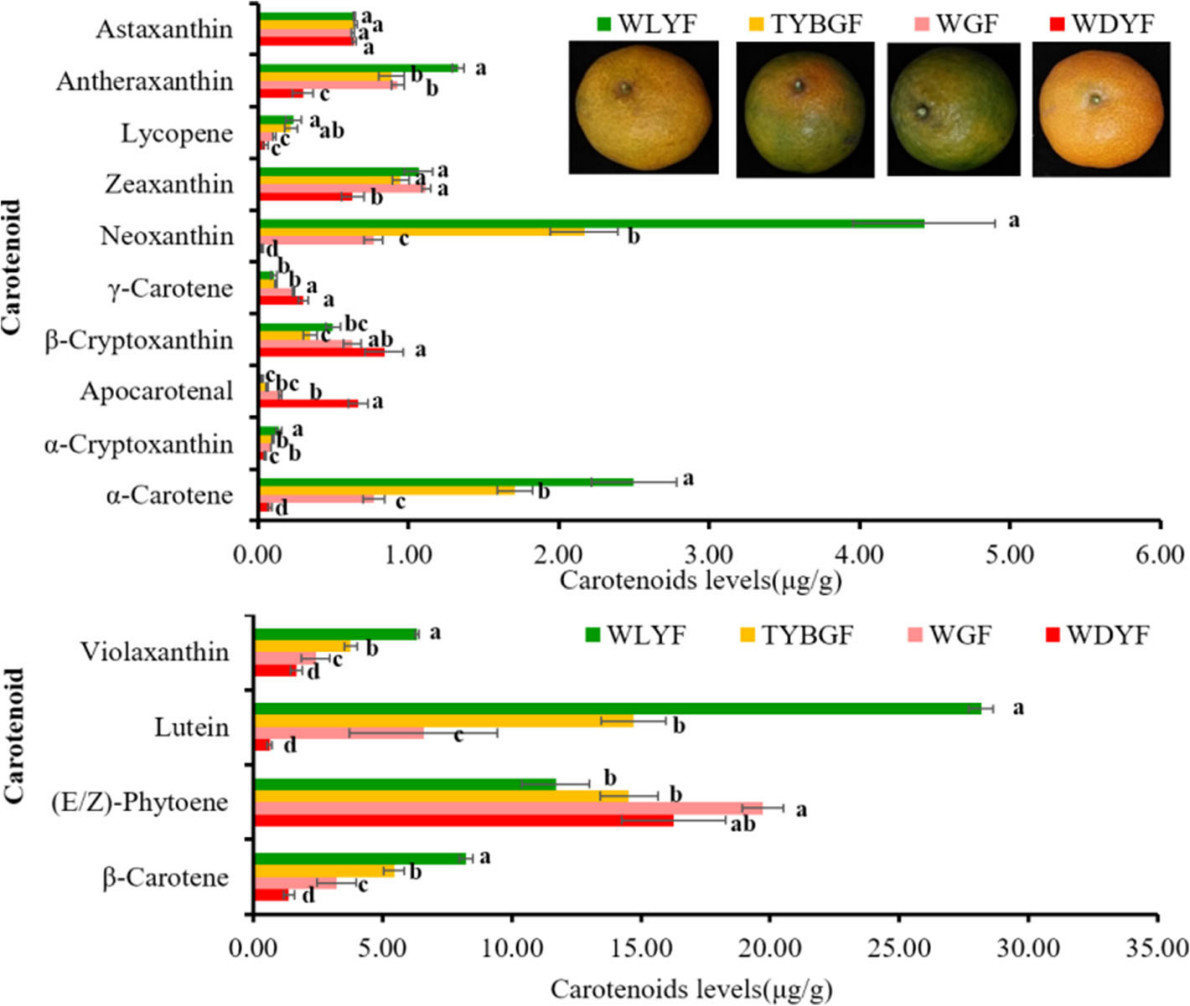
Fourteen kinds of carotenoids were identified in all pericarps

The content of eighteen kinds of carotenoids were identified and detected but fourteen kinds of carotenoids were identified in WGF, TYBGF, WLYF and WDYF fruit pericarps (Supplemental Table S2bcd and Fig. 4). The identified carotenoids didn’t include phytofluene, capsanthin, ε-carotene and capsorubin. The results show that phytoene, lutein, β-carotene, neoxanthin and violaxanthin were the major carotenoid in all samples. Except γ-carotene, phytoene, β-cryptoxanthin, apocarotenal and astaxanthin, the content of other nine kinds of carotenoids in WGF and TYBGF fruits were significantly higher than those in WDYF samples (Fig. 4). The contents of α-carotene, β-carotene, lutein, neoxanthin, zeaxanthin and antheraxanthin in WLYF samples were significantly higher than those in WDYF samples. There was no significant difference between the contents of α-cryptoxanthin, β-cryptoxanthin, γ-carotene, phytoene, lycopene and violaxanthin in WLYF samples and that in WDYF. And only apocarotenal in WLYF sample was significantly lower than that in WDYF samples. In addition, astaxanthin did not show any significant difference in all comparisons (Fig. 4).

### Differential accumulation of phenylpropanoids derivatives in the three comparisons

To compare the differences of primary and secondary metabolism between the three treatment groups and the control group (CK), the fruit pericarps were subjected to LC-ESI-MS/MS analysis. In the present study, most of significantly changed metabolites (SCMs) were flavonoids and other phenylpropanoid derivatives, accounting for 35.81 %. According to the Kyoto Encyclopedia of Genes and Genomes (KEGG) analysis, the phenylpropanoid biosynthesis pathway was the most significantly enriched metabolic pathways in all comparisons [27]. The numbers of differently accumulated flavonoids and phenylpropanoid derivatives were 60, 53 and 29 in WGF vs. WDYF, TYBGF vs. WDYF and WLYF vs. WDYF, respectively. They are shown in supplemental table S3, of which 39 (65 %), 43 (81.13 %) and 24 (82.76 %) metabolites were significantly decreased by comparing WGF, TYBGF, and WLYF to WDYF fruit pericarps, respectively.

### Differential expression of genes in the three comparisons

**Fig. 5:**
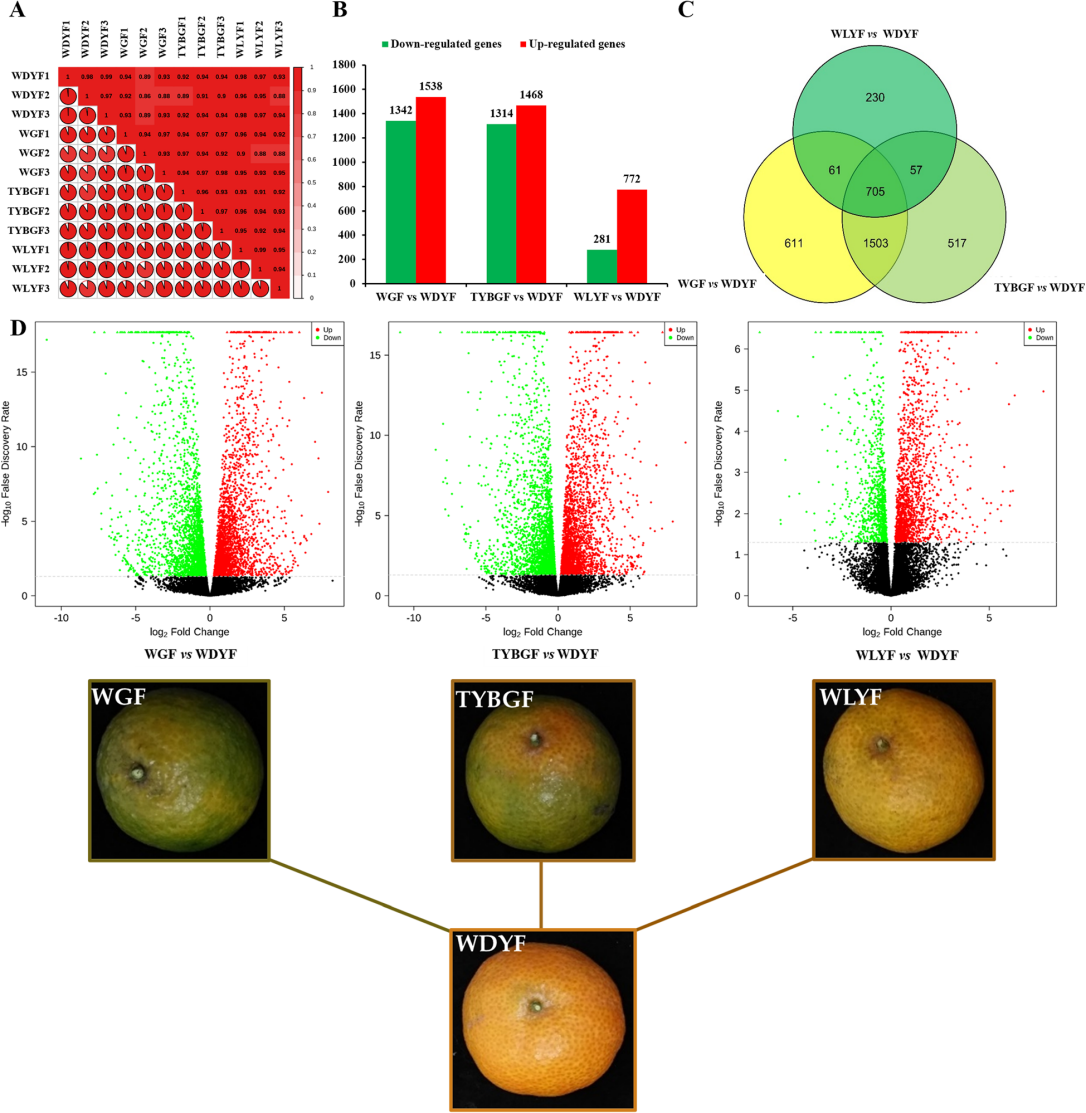
Total down- and up-regulated genes in the three comparisons. **A**: Correlation heatmap of all samples (R^2^ > 0.8); **B**: Status of down/upregulated genes; **C**: Venn diagrams of DEGs in the three comparisons; **D**: Volcano plots of the differentially expressed genes in the three comparisons. Up/down-regulated expression levels of genes are presented by red/green dots, and black dots indicate no difference

To identify genes differentially expressed in the WGF, TYBGF, WLYF fruit pericarps compared with WDYF fruit pericarps (CK), transcriptomic comparison of the four types of materials mentioned above was carried out. RNA-Seq produced 51.74–57.10 (93.41–93.84 %), 47.87–58.56 (93.62–93.83 %), 49.65–55.02 (93.22–93.88) and 47.91–63.47 (93.05–93.61 %) million clean reads from WGF, TYBGF, WLYF and WDYF cDNA libraries after stringent quality checks and data cleanup, respectively (Supplemental Table S4). In total, 44.00-53.48, 40.73–50.03, 42.32–46.86 and 40.68–54.21 million reads were mapped to the *Citrus sinensis* genomic database (https://www.citrusgenomedb.org/organism/Citrus/sinensis. ), with match ratios in the ranges of 80.16–80.69 %, 80.30-80.84 %, 80.00-80.66 % and 79.88–80.66 % in the WGF, TYBGF, WLYF and WDYF groups, respectively (Supplemental Table S4). 19,117, 19,073, 19,306 and 19,263 genes were detected in the WGF, TYBGF, WLYF and WDYF groups, respectively. A high correlation coefficient (R^2^ > 0.99) of gene expression between biological replicates indicated the effectiveness of the data (Fig. 5 A).

With the filter criteria of |log_2_FoldChange| ≥1 and false discovery rate (FDR) < 0.05, there were 2880, 2782, and 1053 differentially expressed genes (DEGs) detected in the three comparisons, WGF vs. WDYF, TYBGF vs. WDYF and WLYF vs. WDYF, respectively, of which 1538, 1468 and 772 genes were upregulated, and 1342, 1314 and 281 genes were downregulated in the three comparisons: WGF vs. WDYF, TYBGF vs. WDYF, and WLYF vs. WDYF, respectively (Supplemental Tables S5, Fig. 5B and D). Venn diagram analysis shows that there were 705 DEGs that were common to the three comparisons (Fig. 5 C). The majority of DEGs in the all comparisons were up-regulated. The greater phenotypic difference of the sample, the more differentially expressed genes of the sample.

The Gene Ontology (GO) analysis was conducted to investigate the roles of HLB-affected genes in the biological process (BP), cell component (CC) and molecular function (MF) terms. The GO analysis of 2880 DEGs in WGF comparison and 2782 DEGs in TYBGF comparison showed some enrichment of five major biological processes, including cellular process, metabolic process and response to stimulus; and 1053 DEGs in WLYF comparison mainly enriched in metabolic process and cellular process by GO analysis. In the molecular function aspect, most of DEGs in the three comparisons all were enriched in catalytic activity and binding. In the cellular component aspect, those DEGs of the three comparisons all were mainly enriched in five categories including cell part, membrane and organelle (Supplemental Fig. S1). Top 20 remarkably enriched GO terms in respect of CC, MF, and BP enriched in the three comparisons were summarized in supporting information (Supplemental Fig. S2). Briefly, several GO terms were associated with photosynthesis process (e.g., GO:0019684 photosynthesis, light reaction, GO:0009521 photosystem, GO:0009522 photosystem I, GO:0015979 photosynthesis) in the three comparisons. Thus, these GO terms were suggested to play vital roles in the HLB-caused abnormal color of ‘Shatangju’ mandarin fruits.

KEGG analysis (*p*-value < 0.05) revealed that all of these DEGs in all comparisons were mainly enriched in 11 metabolic processes. The top three significantly enriched metabolic pathways include photosynthesis-antenna proteins, steroid hormone biosynthesis and phenylpropanoid biosynthesis in WGF vs. WDYF, and photosynthesis-antenna proteins, phenylpropanoid biosynthesis and photosynthesis in TYBGF vs. WDYF and WLYF vs. WDYF (Supplemental Table S6 and Supplemental Fig. S3ACE). Therefore, two metabolic pathways, photosynthesis-antenna proteins and phenylpropanoid biosynthesis, were enriched in all comparisons. Among these DEGs detected in WGF vs. WDYF, TYBGF vs. WDYF and WLYF vs. WDYF, we found 41, 40 and 23 glycosyltransferase genes, 44, 41 and 5 methyltransferase genes, and 25, 23 and 10 acyltransferase genes that catalyzed the synthesis of different types of flavonoids. We also detected 12, 12 and 4 GST (glutathione-S-transferase) genes, 17, 18 and 5 ABC transporter genes, 37, 38 and 13 MATE (multidrug and toxic compound extrusion) genes, and 1, 1 and 0 SNARE (soluble NSF attachment protein receptor) genes, which might play important roles in transporting flavonoids (Supplemental Fig. S3BDF).

### Integrative analysis of transcriptome and metabolome

We combined the analysis of transcriptome and metabolome data to investigate the association between metabolites and genes involved in the same biosynthesis pathway in ‘Shatangju’ fruit pericarps. Results showed that *p*-values of metabolites and genes involved in phenylpropanoid biosynthesis both were less than 0.01 (Supplemental Fig. S4) in the three comparisons. To understand the regulatory mechanism of decreased flavonoids and other phenylpropanoid biosynthesis in the WGF, TYBGF and WLYF fruit pericarps, 60, 53, and 29 SCMs (31, 27 and 11 flavonoids and 29, 26 and 18 other phenylpropanoid-derived metabolites, Supplemental Table S3) and 92, 92, and 44 DEGs (28, 29 and 13 DEGs involving flavonoid biosynthesis and 48, 47 and 23 DEGs involving other phenylpropanoid-derived metabolites, Supplemental Table S7) were subjected to analysis using Pearson’s Correlation Coefficient. Results showed strong positive or significant negative correlations (*R* > 0.8 or <-0.8 and *p*-value < 0.05) between the SCMs and DEGs (Supplemental Table S8). Based on these results, the interaction networks between the SCMs and DEGs (*R* > 0.9) were organized in the WGF, TYBGF and WLYF comparisons (Supplemental Fig. S5). At the same time, the nine-quadrant model was proposed to delineate the relationship of the SCMs with DEGs in the three comparisons. According to the nine-quadrant analysis, the significantly changed flavonoids and other phenylpropanoid-derived SCMs and DEGs (*R* > 0.8 or <-0.8) of the associated metabolic pathways were focused mainly in the 1, 3, 7 and 9 quadrants (Supplemental Table S8, Supplemental Fig. S6). The 3 and 7 quadrants indicate that differential patterns of SCMs and DEGs were consistent, but the 1 and 9 quadrants indicate the inconsistency.

Flavonoids play many different roles throughout the plant’s life cycle. In this study, a large number of flavonoids were identified and quantified in the WGF, TYBGF, WLYF and WDYF fruit pericarps (Supplemental Table S3). In the three comparisons, fifteen DEGs were annotated in the flavonoid biosynthesis pathway in citrus (Supplemental Table S7). In flavonoids, most SCMs were markedly decreased in WGF, TYBGF and WLYF fruit pericarps relative to those in WDYF fruit pericarps, including acetyl-eriodictyol-O-hexoside (-5.52), tricin-4’-O-β-guaiacylglycerol (-15.08), tricin-O- sinapic acid (-3.59), myricetin (-13.67), and genistein (4’,5,7-trihydroxyisoflavone,-14.22) in WGF vs. WDYF; acetyl-eriodictyol-O-hexoside (-4.28), C-hexosyl-apigenin O-caffeoylhexoside (-2.22), tricin 4’-O- β-guaiacylglycerol (-4.90), tricin O-sinapic acid (-2.60), and myricetin (-13.67) in TYBGF vs. WDYF; and acetyl-eriodictyol O-hexoside (-2.57), tricin 4’-O-β-guaiacylglycerol (-2.93), and myricetin (-13.67) in WLYF vs. WDYF (Supplemental Table S3).

**Fig. 6:**
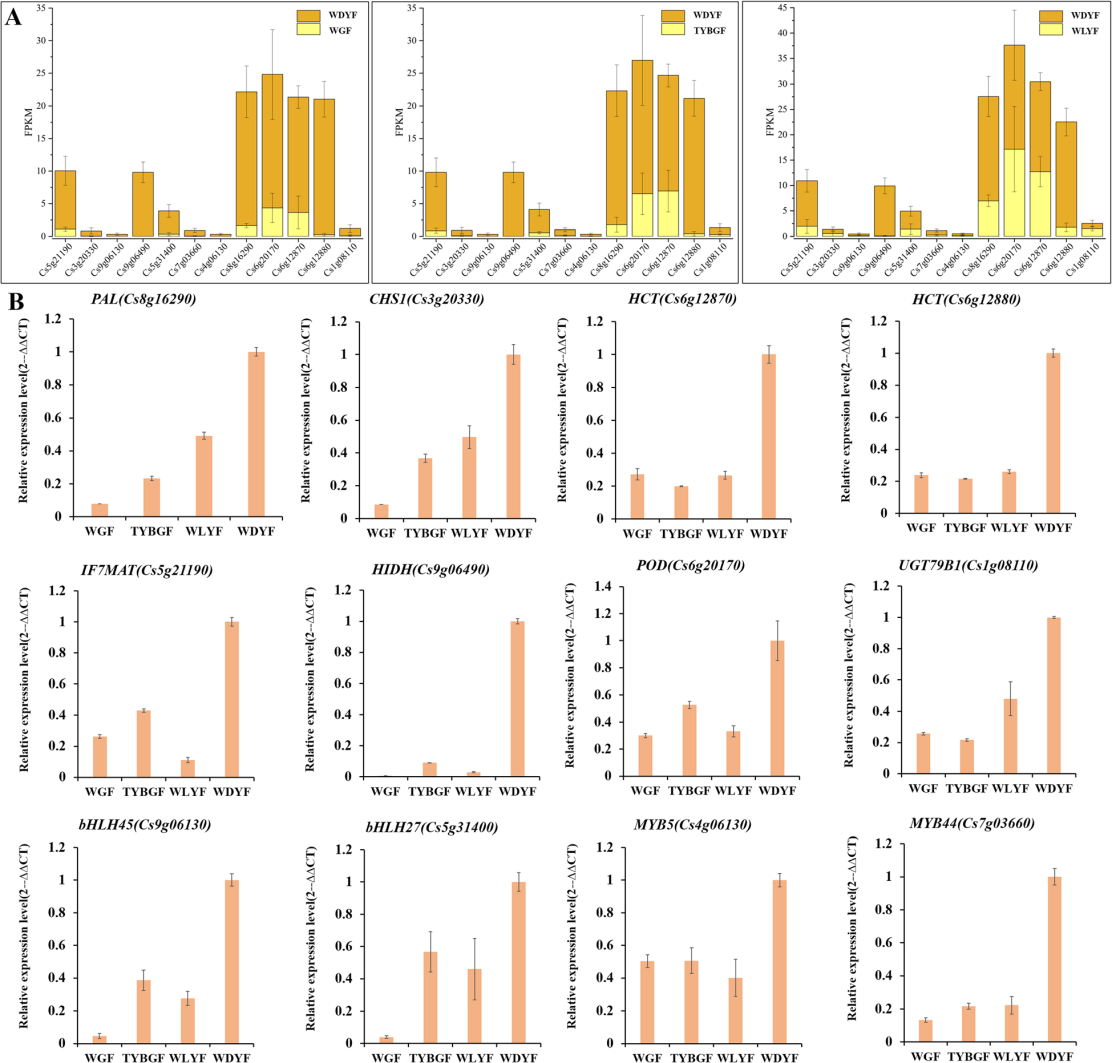
Differentially expressed genes (DEGs) in WGF, TYBGF and WLYF compared with WDYF fruit pericarps **A**: Differentially expressed genes by RNA-Seq; **B**: qRT-PCR showing the expression levels of 12 flavonoids and phenylpropanoid-related DEGs and DETFs in the three comparisons

Six DEGs (the homologs of *CHS*, *HCT* (hydroxy cinnamyl transferase), *HIDH* (2-hydroxyisoflavanone dehydratase), *IF7MAT* (isoflavone 7-O-glucoside-6’’-O-malonyl transferase) and *UGT79B1* genes were screened to validate the transcriptomic results. The qRT-PCR results showed that the expression level of a homologs of *CHS* (*Cs3g20330*), two homologs of *HCT* (*Cs6g12870* and *Cs6g12880*), a homolog of IF7MAT (*Cs5g21190*) a homolog of *HIDH* (*Cs9g06490*) and a homolog of *UGT79B1* (*Cs1g08110*) were lower in WGF, TYBGF and WLYF fruit pericarps than that in WDYF fruit pericarps (Fig. 6). These results were consistent with the RNA-Seq data. Most homolog genes of the flavonoid biosynthesis pathway in citrus showed lower expression levels in WGF, TYBGF and WLYF than that in WDYF fruit pericarps, implying vital function of these enzyme genes in citrus flavonoid biosynthesis.

The metabolome analysis revealed that most of the other phenylpropanoid-derived metabolites in the phenylpropanoid metabolic branches, including coumarin derivatives, coumaraldehyde, coniferyl alcohol, methoxy cinnamaldehyde and cinnamaldehyde, showed significantly lower abundances in WGF, TYBGF and WLYF than in WDYF fruit pericarps (Supplemental Table S3). For instance, 6,7-dimethoxy-4-methylcoumarin (-13.77), p-coumaraldehyde (-5.66) and coniferyl alcohol (-4.48) in WGF vs. WDYF; 6,7-dimethoxy-4-methylcoumarin (-13.77), coniferin (-3.33) and 4-hydroxy-3-methoxycinnamaldehyde (-3.30) in TYBGF vs. WDYF; and isoimperatorin (-3.34), 4-hydroxy-3-methoxycinnamaldehyde (-3.26) and p-coumaraldehyde (-3.17) in WLYF vs. WDYF. Twenty-six DEGs were annotated in the citrus phenylpropanoid biosynthesis pathway (Supplemental Table S7). Some of them, such as *PAL*, *CAD* (cinnamyl-alcohol dehydrogenase), *C3H* (coumarate-3-hydroxylase) and *CCoAOMT* (caffeoyl-CoA-3-O-methyltransferase) homologs, show low expression levels in WGF, TYBGF and WLYF samples. Whereas *CCR* (cinnamoyl-CoA reductase), *COMT* (catechol-o-methyl transferase), *F6H1* (feruloyl-CoA-6-hydroxylase) and *UGT72E* (coniferyl-alcohol glucosyltransferase) homologous genes showed high expression levels in WGF, TYBGF and WLYF samples. These results indicated a complex phenylpropane biosynthesis network. Two DEGs, *Cs8g16290 and Cs6g20170*, were screened to validate the transcriptomic results. The qRT-PCR results showed that the expression level of a homologs of *PAL* (*Cs8g16290*) and a homolog of peroxidase gene (*POD*, *Cs6g20170*) were lower in WGF, TYBGF and WLYF fruit pericarps than that in WDYF fruit pericarps (Fig. 6). These results were consistent with the RNA-Seq data.

**Fig. 7:**
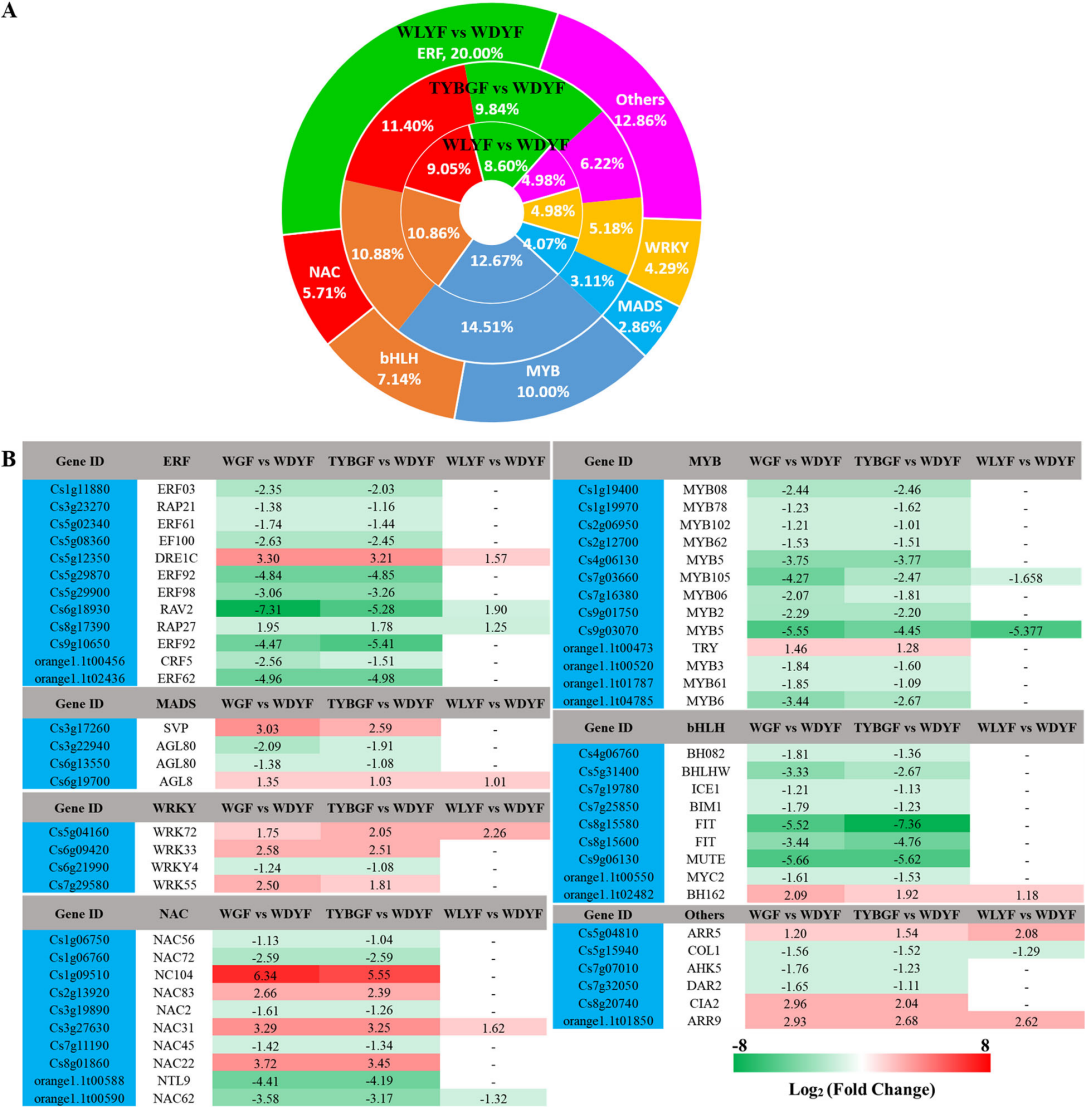
Differentially expressed transcription factors (DETFs) in the three comparisons. **A**: the family classification of DETFs; **B**: the significantly differential expression of TFs; red indicates up-regulated genes and green indicates down-regulated (green) genes

### Transcription factors are correlated with the differential accumulation of metabolites

Transcription factors (TFs), such as those in the MYB and basic helix-loop-helix (bHLHs) families, play key roles in regulating the expression of genes involved in flavonoid biosynthesis in *Arabidopsis* [36–40]. In total, 121, 117 and 43 differentially expressed transcription factors (DETFs) that belong to six TF families were identified in WGF, TYBGF and WLYF comparisons (Fig. 7, Supplemental Table S9). The most abundantly differentially expressed transcription factor families were MYBs (12.67 %) in WGF vs. WDYF, MYBs (14.51 %) in TYBGF vs. WDYF and ERFs (20.00 %) in WLYF vs. WDYF. Results showed that except for *DRE1C* (*Cs5g12350*) in the ERF family, *TRY* (*orange1.1t00473*) in the MYB family and *BH162* (*orange1.1t02482*) in the bHLHs family, which were upregulated in the three comparisons, most of ERFs, MYBs and bHLHs TFs in the three comparisons were down-regulated, especially *RAV2* (*Cs6g18930*) in ERFs, *MYB105* (*Cs7g03660*) in MYBs and *bHLHW* (*Cs5g31400*) and *MUTE* (*Cs9g06130*) in bHLHs (Fig. 7. *SVP* (*Cs3g17260*), *AGL8* (*Cs6g19700*), *WRK33* (*Cs6g09420*), *WRK55* (*Cs7g29580*), *NC104* (*Cs1g09510*), *NAC83* (*Cs2g13920*), *NAC31* (*Cs3g27630*), *NAC22* (*Cs8g01860*), *ARR5* (*Cs5g04810*), *CIA* (*Cs8g20740*), and *ARR9* (*orange1.1t01850*) were up-regulated and may have negative regulatory function in fruit color formation. While some of other DETFs in the MADS, WRKY, and NAC families were down-regulated and may have positive regulatory function. To understand the regulatory mechanism of decreased flavonoid and phenylpropanoid biosynthesis in the WGF, TYBGF and WLYF fruit pericarps, 60, 51 and 22 metabolites and 57, 54 and 17 DETFs were subjected to correlation tests in the WGF, TYBGF and WLYF comparisons. The results showed strong positive and negative correlations (*r* > 0.8 or <-0.8 and *p*-value < 0.05) between SCMs and DETFs, and they mainly focused in the 1, 3, 7 and 9 quadrants (Supplemental Table S10, Supplemental Fig. S6).

## Discussion

Carotenoids are a large class of natural lipid-soluble pigments and are important for plant including citrus plant [14]. More than 1000 kinds of carotenoids have been identified from natural sources [15]. Carotenoid pigments massively accumulate in citrus fruit. And their contents and compositions are important indexes for the nutritional and commercial quality [41]. Previous studies have shown that the contents of β-carotenoid, lutein, and α-carotene decreased rapidly to a low level, and massive accumulation of β-cryptoxanthin, zeaxanthin and violaxanthin occurred in some varieties during the ripening process [41]. In this study, we identified and determined the contents of fourteen kinds of carotenoids in the four types of mandarin ‘Shatangju’ fruit pericarps (Supplemental Table S2, Fig. 4). The highest accumulation of phytoene was in the WLYF group, while the lowest accumulation was in the WGF group (Fig. 4). The α-carotene and lutein were significantly higher in WGF, TYBGF and WLYF than those in WDYF samples. The γ-carotene and β-cryptoxanthin, astaxanthin and apocarotenal in the WDYF group were significantly higher than those in the other three groups. This was consistent with previous studies. This indicated that WGF, TYBGF and WLYF has not reached the full maturity stage. Several TFs have been implicated in the regulation of carotenoid accumulation during fruit ripening, including the MADS-box TFs *RIN*, *TAGL1*, and *TDR4* [42–45], the AP2/ERF family TFs *SlAP2a* and *SlERF6* [46, 47] and MYB family TFs *RCP*1 [48]. According to the transcriptome analysis and the correlation analysis between transcription factors and carotenoids, multiple genes, such as AP2/ERF family TFs *RAP27* (Cs8g17390) and NAC family TFs *NAC22* (Cs8g01860), *NAC62* (*orange1.1t00590*) and *NAC45* (*Cs7g11190*), and bHLH family TFs *BHLHW* (*Cs5g31400*), and *BH162* (*Cs2g05960*) and MYB family TFs *MYB36* (*Cs4g13170*) (Supplemental Table S11). These data indicate that these TFs may contribute to the synthesis of carotenoids and be affected by the invasion of HLB. This aspect should be further studied to confirm the effect mechanism.

Carotenoids and flavonoids both are important factors for the pigment of citrus fruits. The transcriptional level of flavonoid related genes was regulated by transcription factors (TFs), such as MYB-bHLH-WD40 (MBW) complex [39, 48, 49], WRKY [50] and MADS [51]. MYB transcription factor is a decisive regulator in the MBW complex. It positively or negatively regulates structural genes in the flavonoid pathway and maintains the balance of flavonoids in plant organs. The *MdMYB1* in apple [52], *PpMYB10* in peach [53] and *VvMYBA1*, *VvMYBA2*, *VvMYBA-pa2* in grape [54, 55] belong to the positive regulation of MYB factors and improve the transcription level of flavonoid related genes. *FaMYB9/FaMYB11* [56] and *FaMYB1* in strawberry [57], *PpMYB18* in peach [58], *Vvmybc2-l3* and *Vvmybc2-l2* in grape [59], *MdMYB16* and *MdMYB15L* in apple [60, 61] were negative regulation of MYB factors. They inhibit the synthesis of anthocyanins. In the present study, the transcriptome profiles of abnormal pigment fruit pericarps and WDYF (CK) fruit pericarps were analyzed. And it was found that some TF families, including MADS, WRKY, MYB and bHLHs, showed significantly differential expression levels. As such, 66.67 % 80.77 and 66.67 % of MYBs were significantly down-regulated in WGF, TYBGF and WLYF comparisons. All of the differentially expressed TFs may be candidate genes that regulated flavonoid biosynthesis in citrus.

Transcriptomics integrated with metabolite analysis revealed that most flavonoids and other phenylpropanoid-derived metabolites and some carotenoids (phytoene, γ-carotene, β-cryptoxanthin, astaxanthin and apocarotenal) showed drastically lower abundances in WGF, TYBGF and WLYF than in WDYF (CK) fruits. Based on chemical structures of the flavonoids and other phenylpropanoid-derived metabolites, the putative roadmaps of flavonoids and other phenylpropanoid biosynthesis pathways were drawn. The levels of three flavanones (butein, eriodictyol and phloretin), the common precursors of flavonoids, were decreased in WGF vs. WDYF, especially eriodictyol in all comparisons. It should be noted that five metabolites (6,7-dimethoxy-4-methylcoumarin, acetyl-eriodictyol o-hexoside, myricetin, tricin 4’-o-β-guaiacylglycerol and p-coumaraldehyde) in all flavonoids and other phenylpropanoid-derived products showed the most obviously decreased abundances in all comparisons, especially myricetin and tricin 4’-o-β-guaiacylglycerol. 6,7-Dimethoxy-4- methyl-coumarin and myricetin are known to have significant antibacterial activity [62]. Therefore, this result may cause WGF, TYBGF and WLYF fruit pericarps to be high in terms of the population of pathogenic bacteria.

## Conclusions

In this study, we explored that the population of pathogenic bacteria followed the order WGF > TYBGF > WLYF > WDYF. In regard to chlorophyll and carotenoid abundance, the WGF had the highest total content of chlorophyll and the lowest total content of carotenoid. WGF and TYBGF fruit pericarps were low in phytoene, γ-carotene, β-cryptoxanthin, astaxanthin and apocarotenal, and the levels of other carotenoids were significantly higher than those in WDYF fruit pericarps. And WLYF was only short of apocarotenal. Integrative analysis of transcriptomic and metabolomic data indicated that the influencing mechanisms of HLB infection on the mandarin ‘Shatangju’ fruit pericarps may involve phenylpropanoid-derived metabolic pathways, and extensive down-regulation genes of photosynthetic-related protein. This work not only provides insights into the molecular and biochemical mechanisms of HLB-infection on the mandarin ‘Shatangju’ fruit pericarps but may be of significance in uncovering the unknown gene for responding to HLB.

## Supplementary Information

**Additional file 1: Supplemental Figure S1.** GO analysis of DEGs in three comparisons. **Supplemental Figure S2. **Statistical analysis of GO enrichment of DEGs in the three comparisons. **Supplemental Figure S3.** KEGG analysis of DEGs in the three comparisons. **Supplemental Figure S4. **KEGG analysis of SCMs and DEGs in the three comparisons. **Supplemental Figure S6. **the nine-quadrant analysis of all SCMs and DEGs in the three comparisons.

**Additional file 2: Supplemental Figure S5.** Connection network between regulatory genes and flavonoid and carotenoid-related metabolites.

**Additional file 3: Supplemental Table S1.** Sequences of specific primers for qRT-PCR.

**Additional file 4: Supplemental Table S2.** a: the phenotype and color analysis of citrus fruits (*Citrus reticulata* cv. ‘Shatangju’) at the maturity period. b: UHPLC-MS/MS data of carotenoids from different colored citrus fruit pericarps (*Citrus reticulata* cv. ‘Shatangju’) at the maturity period. c: Carotenoids quantify level from different colored citrus fruits (*Citrus reticulata* cv. ‘Shatangju’) at the maturity period. d: the equation for eighteen kinds of carotenoids.

**Additional file 5: Supplemental Table S3.** the significantly changed flavonoids and phenylpropanoids in WGF, TYBGF and WLYF compared with WDYF.

**Additional file 6: Supplemental Table S4.** Summary of the sequencing and genome comparison.

**Additional file 7: Supplemental Table S5.** The significantly changed genes in WGF, TYBGF and WLYF compared with WDYF.

**Additional file 8: Supplemental Table S6.** KEGG of DEGs in the three comparisons.

**Additional file 9: Supplemental Table S7.** the genes involved in flavonoids and phenylpropanoids metabolism in WGF, TYBGF and WLYF compared with WDYF.

**Additional file 10: Supplemental Table S8.** Pearson correlation coefficient of genes and metabolites involved in the flavonoid metabolic pathway in WGF, TYBGF and WLYF compared with WDYF.

**Additional file 11: Supplemental Table S9.** Differentially expression transcription factors in WGF, TYBGF and WLYF compared with WDYF.

**Additional file 12: Supplemental Table S10.** Pearson correlation coefficient of TFs and metabolites involved in the flavonoid metabolic pathway in WGF, TYBGF and WLYF compared with WDYF.

**Additional file 13: Supplemental Table S11.** Pearson correlation coefficient of genes and carotenoids in WGF, TYBGF and WLYF compared with WDYF.

## Data Availability

All relevant supporting data sets are included in the article and its supplemental files.

## Abbreviations

WGF: Whole Green Fruits
TYBGF: Top-Yellow and Base-Green Fruits
WLYF: Whole Light-Yellow Fruits
WDYF: Whole Dark Yellow Fruits
DEGs: Differentially Expressed Genes
HLB: Huanglongbing
PMFs: O-Poly-Methoxylated Flavones
PAL: Phenylalanine Ammonia-Lyase
C4H: Cinnamate 4-Hydroxylase
4CL: 4-Coumarate Coenzyme A Ligase
CHS: Chalcone Synthase
CHI: Chalcone Isomerase
F3H: Flavanone 3-Hydroxylase
F3’H: Flavonoid 3’-Hydroxylase
IFS: Isoflavone Synthase
FLS: Flavonol Synthase
DFR: Dihydroflavonol-4-Reductase
LAR: Leucoanthocyanidin Reductase
ANR: Anthocyanidin Reductase
ANS: Anthocyanidin Synthase
CI: Color Index
SCMs: Significantly Change Metabolites
FPKM: Reads Per Kilo base per Million mapped reads
FDR: False Discovery Rate
BP: Biological Process
CC: Cell Component
MF: Molecular Functional
GO: Gene Ontology
KEGG: Kyoto Encyclopedia of Genes and Genomes
GST: Glutathione S-transferase
MATE: Multidrug and Toxic Compound Extrusion
SNARE: Soluble NSF Attachment Protein Receptor
HCT: Hydroxy Cinnamoyl Transferase
HIDH: 2-Hydroxyisoflavanone Dehydratase
IF7MAT: Isoflavone 7-O-Glucoside-6’-O-Malonyltransferase
UGT: UDP-Glycosyltransferases
CAD: Cinnamyl Alcohol Dehydrogenase
C3H: Coumarate-3-Hydroxylase
CCoAOMT: Caffeoyl-Coa-3-O-Methyltransferase
CCR: Cinnamoyl Coa Reductase
COMT: Catechol-O-Methyl Transferase
F6H1: Feruloyl-Coa-6-Hydroxylase
POD: Peroxidase
bHLH: Basic Helix-Loop-Helix
CAN: Acetonitrile
UHPLC: Ultra-Performance Liquid Chromatography
BHT: 2;6-Di-Tert-Butyl-4-Methylphenol
CUR: Curtain Gas
CAD: Collision Gas
DP: Declustering Potential
CE: Collision Energy
VIP: Variable Importance in the Projection
