# Supplementary material for: Integrative analysis of metabolome and transcriptome profiles provides insight into the fruit pericarp pigmentation disorder caused by ‘Candidatus Liberibacter asiaticus’ infection
Source: BMC Plant Biol. 2021 Aug 25;21:397. doi: 10.1186/s12870-021-03167-3 (PMC8385863; doi:10.1186/s12870-021-03167-3)
Supplement: Supplementary file 1 — Additional file 1: Supplemental Figure S1. GO analysis of DEGs in three comparisons. Supplemental Figure S2. Statistical analysis of GO enrichment of DEGs in the three comparisons. Supplemental Figure S3. KEGG analysis of DEGs in the three comparisons. Supplemental Figure S4. KEGG analysis of SCMs and DEGs in the three comparisons. Supplemental Figure S6. the nine-quadrant analysis of all SCMs and DEGs in the three comparisons. [file 12870_2021_3167_MOESM1_ESM.docx]

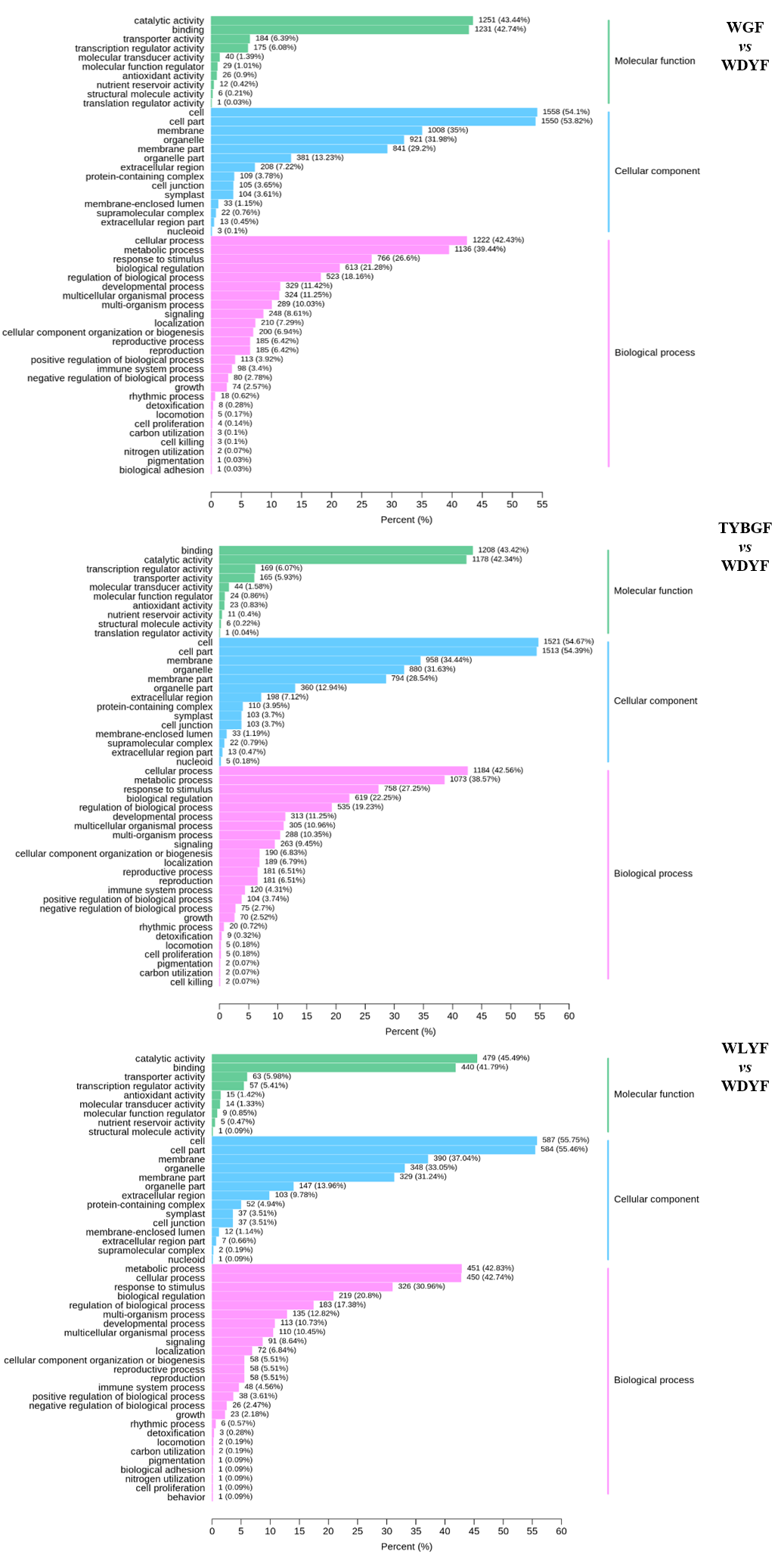


**Supplemental Fig. S1** GO analysis of DEGs in the three comparisons


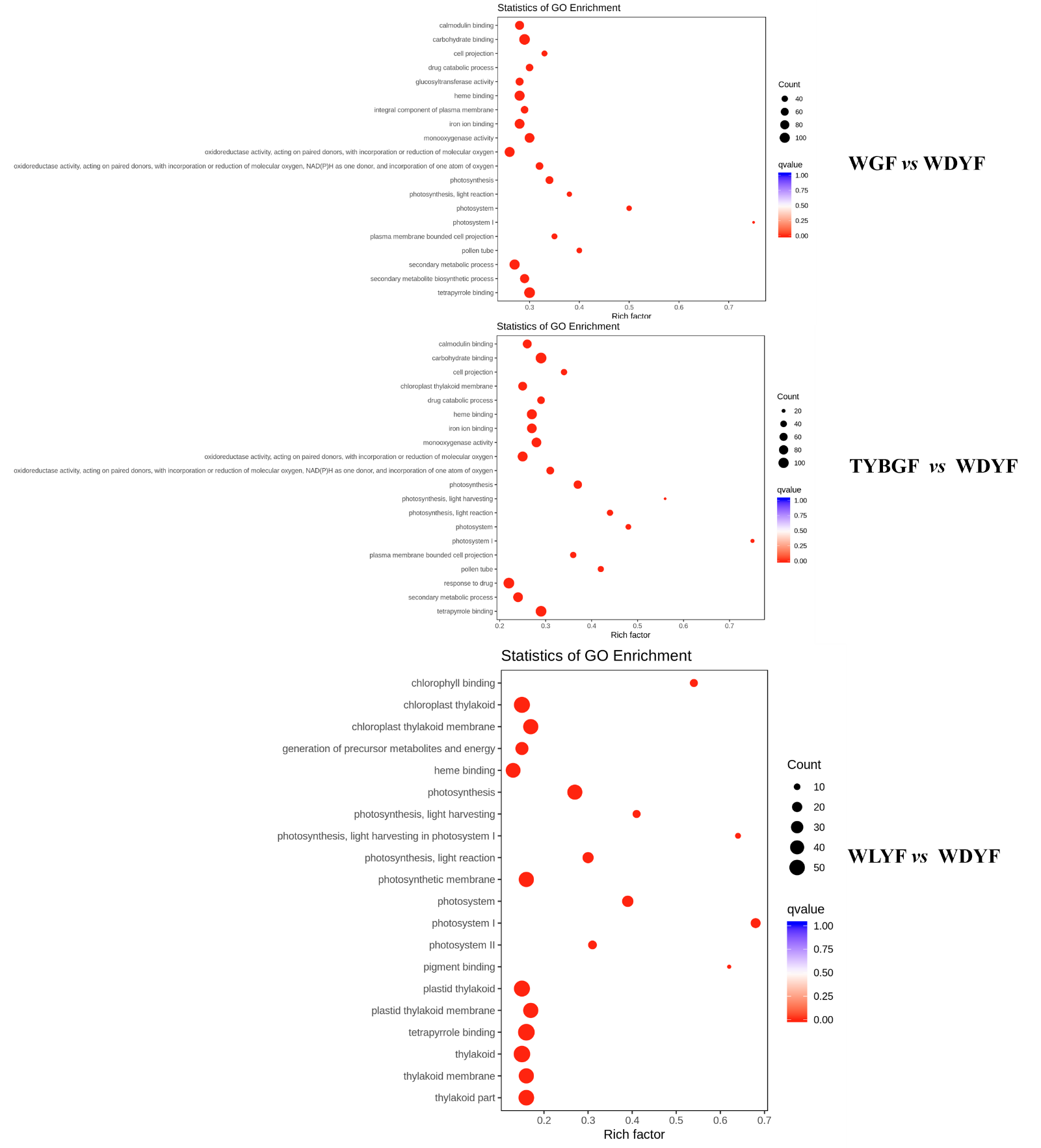


**Supplemental Fig. S2** Statistical analysis of GO enrichment of DEGs in the three comparisons


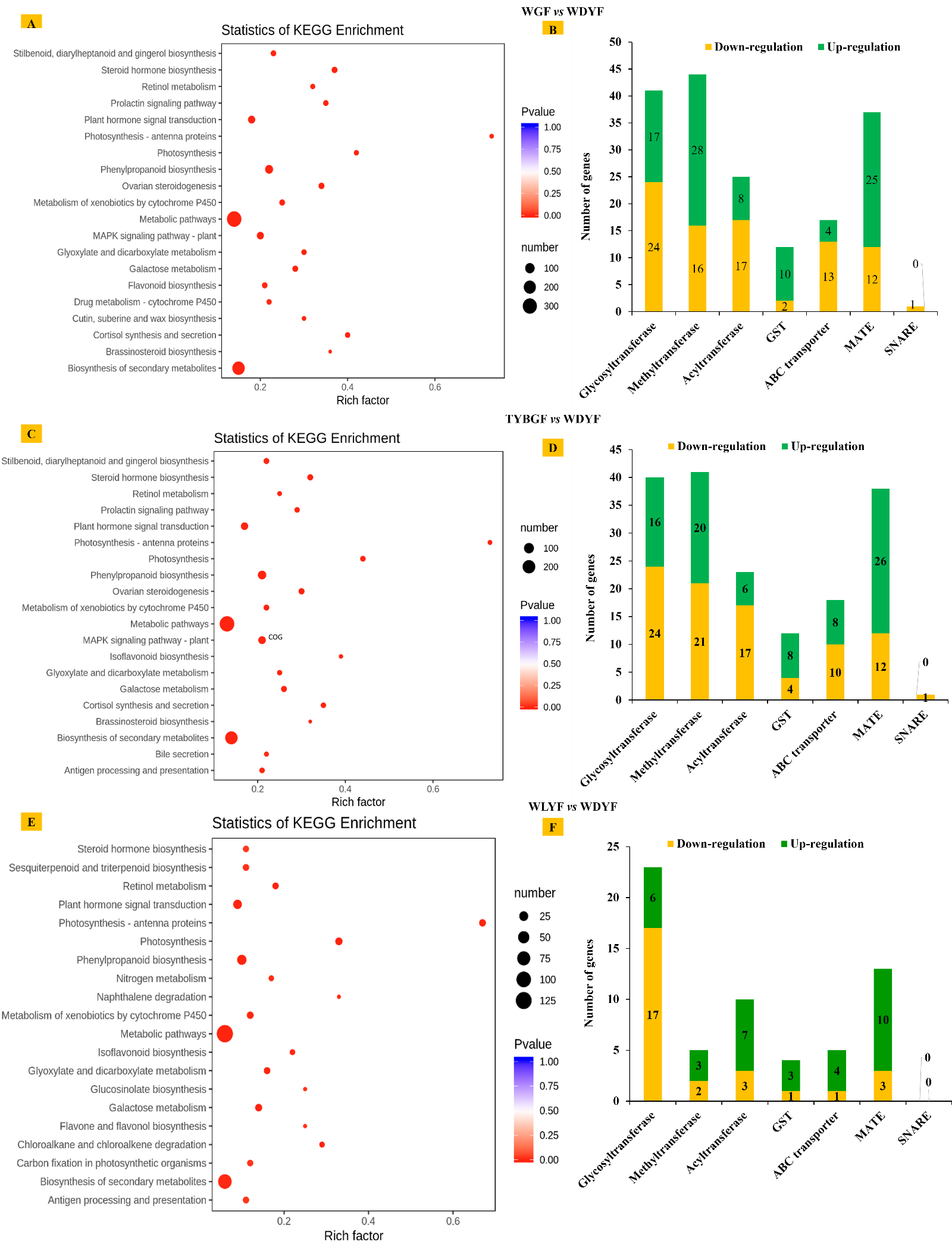


**Supplemental Fig. S3** KEGG analysis of DEGs in the three comparisons


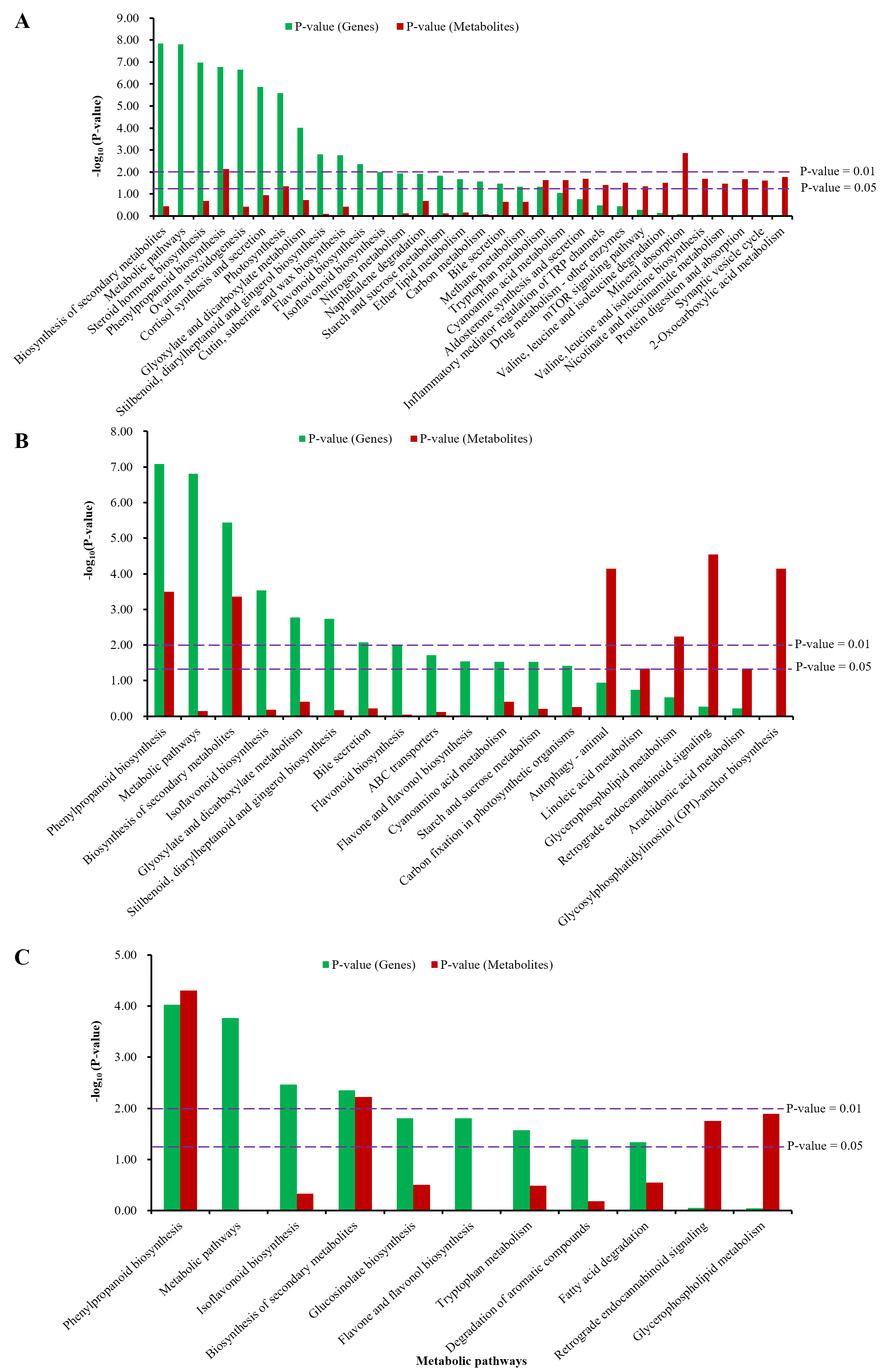


**Supplemental Fig. S4** KEGG analysis of SCMs and DEGs in the three comparisons

A: WGF *vs.* WDYF; B: TYBGF *vs.* WDYF; C: WLYF *vs.* WDYF


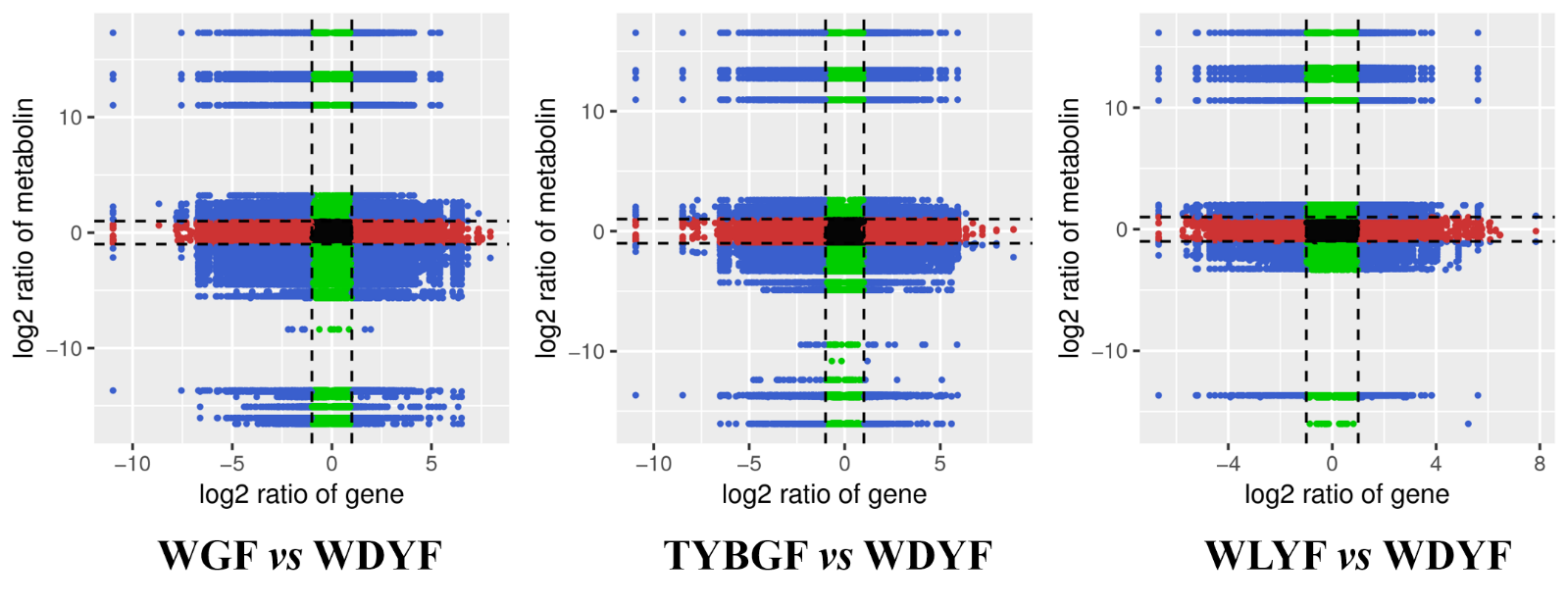


**Supplemental Fig. S5** the nine-quadrant analysis of all SCMs and DEGs in three comparisons

**Supplementary Fig. S6** Connection network between regulatory genes and flavonoid and carotenoid-related metabolites

A: WGF *vs.* WDYF; B: TYBGF *vs.* WDYF; C: WLYF *vs.* WDYF

The networks in WGF *vs.* WDYF (A), TYBGF *vs.* WDYF(B) and WLYF *vs.* WDYF(C) were visualized with the Cytoscape software (version 3.8.0). Acid Methyl Ester: Trans-4-Hydroxycinnamic Acid Methyl Ester; Astragalin: Kaempferol 3-O-Glucoside (Astragalin); Coumarin: O-Feruloyl Coumarin; Cynaroside: Luteolin 7-O-Glucoside (Cynaroside); Glucopyranosyl Sinapate: 1-O-Beta-D-Glucopyranosyl Sinapate; Glucoside Chloride: Peonidin 3-O-Glucoside Chloride; Guaiacylglycerol: Tricin 4'-O-Β-Guaiacylglycerol; Hexoside: Acetyl-Eriodictyol O-Hexoside; Hydroxylcoumarin: O-Feruloyl 2-Hydroxylcoumarin; Isorhoifolin: Apigenin 7-Rutinoside (Isorhoifolin); Isosakuranetin: Isosakuranetin (4'-Methylnaringenin); Keracyanin: Cyanidin 3-O-Rutinoside (Keracyanin); Methoxycinnamaldehyde: 4-Hydroxy-3-Methoxycinnamaldehyde; Methoxycinnamate: Hydroxy-Methoxycinnamate; Methylcoumarin: 6,7-Dimethoxy-4-Methylcoumarin; Methylquercetin: 3,7-Di-O-Methylquercetin; P-Coumaryl Alcohol: P-Coumaryl Alcohol; Pentoside: Luteolin O-Hexosyl-O-Pentoside; Peonidin: Peonidin O-Hexoside; Quinic Acid: 6,7-Dihydroxycoumarin 6-O-Quinic Acid; Rhamnetin: Rhamnetin (7-O-Methxyl Quercetin); Rhoifolin: Apigenin 7-O-Neohesperidoside (Rhoifolin); Sinapic Acid: Tricin O-Sinapic Acid; Sinapoylhexoside: 6-C-Hexosyl-Apigenin O-Sinapoylhexoside; Propionic Acid: 3-(4-Hydroxyphenyl) Propionic Acid; Genistein: Genistein (4',5,7-Trihydroxyisoflavone).
