# Supplementary figures and images for: Integrative analysis of metabolome and transcriptome profiles provides insight into the fruit pericarp pigmentation disorder caused by ‘Candidatus Liberibacter asiaticus’ infection
Source: BMC Plant Biol. 2021 Aug 25;21:397. doi: 10.1186/s12870-021-03167-3 (PMC8385863; doi:10.1186/s12870-021-03167-3)

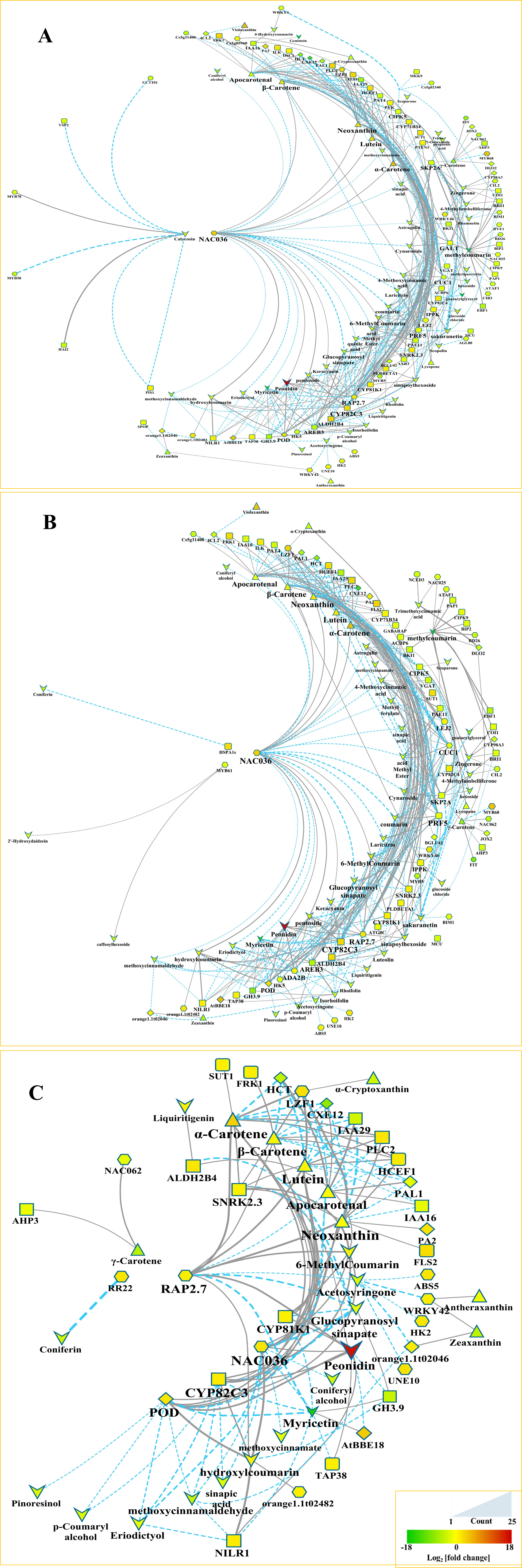

Supplement: Supplementary file 2 — Additional file 2: Supplemental Figure S5. Connection network between regulatory genes and flavonoid and carotenoid-related metabolites. [file 12870_2021_3167_MOESM2_ESM.png]
